# *catena*-Poly[[diaqua­calcium(II)]-bis­(μ-quinoline-3-carboxyl­ato)]

**DOI:** 10.1107/S1600536810039401

**Published:** 2010-10-23

**Authors:** Dong-Liang Miao, Shi-Jie Li, Wen-Dong Song, Juan-Hua Liu, Xiao-Fei Li

**Affiliations:** a College of Food Science and Technology, Guangdong Ocean University, Zhanjiang 524088, People’s Republic of China; b College of Science, Guangdong Ocean University, Zhanjiang 524088, People’s Republic of China

## Abstract

In the title complex, [Ca(C_10_H_6_NO_2_)_2_(H_2_O)_2_]_*n*_, the Ca^II^ ion is eight-coordinated by six carboxyl­ate O atoms from four separate quinoline-3-carboxyl­ate ligands, two of which are bidentate chelate and two bridging, and two water mol­ecules in a distorted square-anti­prismatic geometry. The bridging groups form a polymeric chain substructure extending along the *c* axis, the chains being connected by coordinated-water O—H⋯N and O—H⋯O_carboxyl­ate_ hydrogen bonds into a three-dimensional framework structure.

## Related literature

For the potential uses and diverse structural types of metal complexes with the quinoline-3-carboxyl­ate ligand, see: Hu *et al.* (2007 ▶). For related structures, see: Martell & Smith (1974 ▶); Haendler (1986 ▶, 1996 ▶); Okabe & Koizumi (1997 ▶); Okabe & Makino (1998 ▶, 1999 ▶); Okabe & Muranishi (2002 ▶); Odoko *et al.* (2001 ▶).
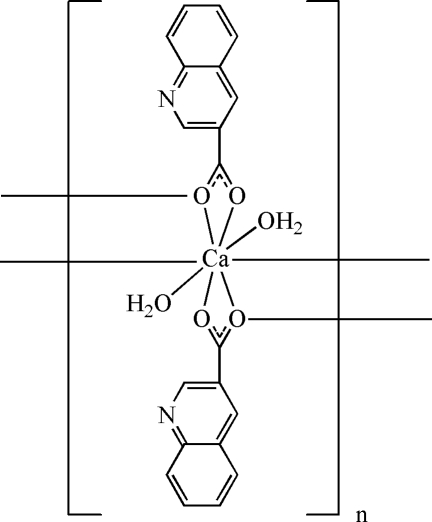


## Experimental

### 

#### Crystal data

[Ca(C_10_H_6_NO_2_)_2_(H_2_O)_2_]
*M* *_r_* = 420.43
Monoclinic, 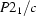

*a* = 16.0115 (16) Å
*b* = 15.3636 (16) Å
*c* = 7.7962 (8) Å
β = 97.928 (1)°
*V* = 1899.5 (3) Å^3^
*Z* = 4
Mo *K*α radiation
μ = 0.37 mm^−1^
*T* = 296 K
0.30 × 0.26 × 0.25 mm

#### Data collection

Bruker APEXII area-detector diffractometer
Absorption correction: multi-scan (*SADABS*; Bruker, 2008 ▶) *T*_min_ = 0.897, *T*_max_ = 0.913
9735 measured reflections
3411 independent reflections
2433 reflections with *I* > 2σ(*I*)
*R*_int_ = 0.032

#### Refinement

*R*[*F* ^2^ > 2σ(*F* ^2^)] = 0.035
*wR*(*F* ^2^) = 0.090
*S* = 1.02
3411 reflections
262 parameters
6 restraints
H-atom parameters constrained
Δρ_max_ = 0.25 e Å^−3^
Δρ_min_ = −0.26 e Å^−3^

### 

Data collection: *APEX2* (Bruker, 2008 ▶); cell refinement: *SAINT* (Bruker, 2008 ▶); data reduction: *SAINT*; program(s) used to solve structure: *SHELXS97* (Sheldrick, 2008 ▶); program(s) used to refine structure: *SHELXL97* (Sheldrick, 2008 ▶); molecular graphics: *SHELXTL* (Sheldrick, 2008 ▶); software used to prepare material for publication: *SHELXTL*.

## Supplementary Material

Crystal structure: contains datablocks I, global. DOI: 10.1107/S1600536810039401/zs2067sup1.cif

Structure factors: contains datablocks I. DOI: 10.1107/S1600536810039401/zs2067Isup2.hkl

Additional supplementary materials:  crystallographic information; 3D view; checkCIF report

## Figures and Tables

**Table 1:** Hydrogen-bond geometry (Å, °)

*D*—H⋯*A*	*D*—H	H⋯*A*	*D*⋯*A*	*D*—H⋯*A*
O2*W*—H4*W*⋯N2^i^	0.88	2.01	2.880 (3)	170
O2*W*—H3*W*⋯O1^ii^	0.92	1.92	2.813 (2)	163
O1*W*—H2*W*⋯N1^iii^	0.72	2.18	2.885 (2)	165
O1*W*—H1*W*⋯O4^iv^	0.84	1.94	2.785 (2)	174

Symmetry codes: (i) 
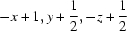
; (ii) 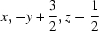; (iii) 
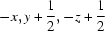
; (iv) 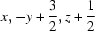.

**Fig. 1.**
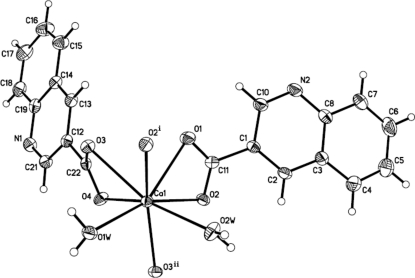
The structure of the title compound, showing the atomic numbering scheme. Non-H atoms are shown with 30% probability displacement ellipsoids.

**Fig. 2.**
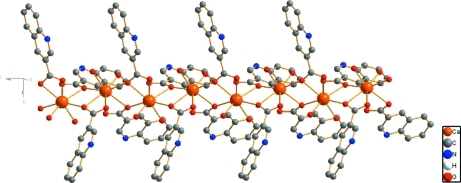
The one-dimensional chain substructure of (I) extending along c.

**Fig. 3.**
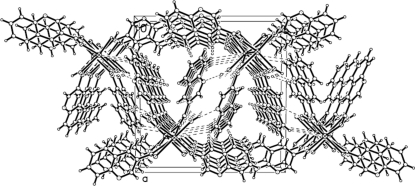
The three-dimensional hydrogen-bonded structure of (I).

## supplementary crystallographic information

### Comment

Design and synthesis of metal-organic complexes have attracted extensive attention in coordination chemistry. Quinoline-2-carboxylic acid, which is a tryptophan metabolite (Martell & Smith, 1974) can be considered as a potential ligand and the crystal structures of a number of metal complexes containing the quinoline-2-carboxylate ligand have been determined, e.g. with Mn^II^ (Haendler, 1996; Okabe & Koizumi, 1997), Cu^II^ (Haendler, 1986), V^IV^ (Okabe & Muranishi, 2002). Fe^II^ and Co^II^ (Okabe & Makino, 1998, 1999), and Ni^II^ (Odoko *et al.*, 2001). However, to the best of our knowledge, there are few crystal structures containing the quinoline-3-carboxylate ligand, one example being the coordination polymer with Zn^II^ (Hu *et al.*,2007). In this paper, we report the synthesis and structure of a new Ca^II^ complex obtained from the reaction of quinoline-3-carboxylic acid with CaCl_2_ under hydrothermal condition, the title compound [Ca(C_20_H_12_N_2_O_4_)(H_2_O)_2_]_n_ (I).

In the title complex molecule the Ca^II^ atom is eight-coordinated by six carboxylate O atoms from four separate quinoline-2-carboxylate ligands (two bidentate chelate and two bridging) and two water O atoms, in a distorted square-antiprismatic environment (Fig. 1). The bridging carboxylate O atoms (O2 and O3) [Ca—O, 2.3877 (16), 2.3829 (16) Å] link separate Ca^II^ centres forming a one-dimensional chain substructure extended along *c* (Fig.2). The chains are inter-connected by coordinated-water O—H···N and O—H···O_carboxylate_ hydrogen bonds (Table 1) giving a three-dimensional framework structure (Fig.3).

### Experimental

A mixture of CaCl_2_ (0.02 g, 0.2 mmol) and quinoline-3-carboxylic acid (0.04 g, 0.2 mmol) in 12 ml of distilled water was sealed in an autoclave equipped with a Teflon liner (20 ml) and then heated at 394 K for 2 days. Crystals of the title compound were obtained by slow evaporation of the solvent at room temperature.

### Refinement

Water H atoms were located in a difference Fourier map and were allowed to ride on the parent atom, with *U*_iso_(H) = 1.5*U*_eq_(O). Other H atoms were placed at calculated positions and were treated as riding on parent atoms with C—H = 0.96 Å and N—H = 0.86 Å and *U*_iso_(H) = 1.2 or 1.5*U*_eq_(C, N).

### Crystal data

**Table:**

[Ca(C_10_H_6_NO_2_)_2_(H_2_O)_2_]	*F*(000) = 872
*M**_r_* = 420.43	*D*_x_ = 1.470 Mg m^−^^3^
Monoclinic, *P*2_1_/*c*	Mo *K*α radiation, λ = 0.71073 Å
Hall symbol: -P 2ybc	Cell parameters from 3600 reflections
*a* = 16.0115 (16) Å	θ = 1.4–25.0°
*b* = 15.3636 (16) Å	µ = 0.37 mm^−^^1^
*c* = 7.7962 (8) Å	*T* = 296 K
β = 97.928 (1)°	Block, colorless
*V* = 1899.5 (3) Å^3^	0.30 × 0.26 × 0.25 mm
*Z* = 4	

### Data collection

**Table:**

Bruker APEXII area-detector diffractometer	3411 independent reflections
Radiation source: fine-focus sealed tube	2433 reflections with *I* > 2σ(*I*)
graphite	*R*_int_ = 0.032
φ and ω scan	θ_max_ = 25.2°, θ_min_ = 1.9°
Absorption correction: multi-scan (*SADABS*; Bruker, 2008)	*h* = −19→16
*T*_min_ = 0.897, *T*_max_ = 0.913	*k* = −18→18
9735 measured reflections	*l* = −9→9

### Refinement

**Table:**

Refinement on *F*^2^	Primary atom site location: structure-invariant direct methods
Least-squares matrix: full	Secondary atom site location: difference Fourier map
*R*[*F*^2^ > 2σ(*F*^2^)] = 0.035	Hydrogen site location: inferred from neighbouring sites
*wR*(*F*^2^) = 0.090	H-atom parameters constrained
*S* = 1.02	*w* = 1/[σ^2^(*F*_o_^2^) + (0.0344*P*)^2^ + 0.6204*P*] where *P* = (*F*_o_^2^ + 2*F*_c_^2^)/3
3411 reflections	(Δ/σ)_max_ < 0.001
262 parameters	Δρ_max_ = 0.25 e Å^−^^3^
6 restraints	Δρ_min_ = −0.26 e Å^−^^3^

### Special details

**Table:**

Geometry. All e.s.d.'s (except the e.s.d. in the dihedral angle between two l.s. planes) are estimated using the full covariance matrix. The cell e.s.d.'s are taken into account individually in the estimation of e.s.d.'s in distances, angles and torsion angles; correlations between e.s.d.'s in cell parameters are only used when they are defined by crystal symmetry. An approximate (isotropic) treatment of cell e.s.d.'s is used for estimating e.s.d.'s involving l.s. planes.
Refinement. Refinement of *F*^2^ against ALL reflections. The weighted *R*-factor *wR* and goodness of fit *S* are based on *F*^2^, conventional *R*-factors *R* are based on *F*, with *F* set to zero for negative *F*^2^. The threshold expression of *F*^2^ > σ(*F*^2^) is used only for calculating *R*-factors(gt) *etc*. and is not relevant to the choice of reflections for refinement. *R*-factors based on *F*^2^ are statistically about twice as large as those based on *F*, and *R*- factors based on ALL data will be even larger.

### Fractional atomic coordinates and isotropic or equivalent isotropic displacement parameters (Å^2^)

**Table:**

	*x*	*y*	*z*	*U*_iso_*/*U*_eq_	
Ca1	0.24944 (3)	0.78080 (3)	0.25589 (6)	0.02366 (14)	
C1	0.45779 (14)	0.63295 (14)	0.1201 (3)	0.0284 (5)	
N2	0.58144 (13)	0.54488 (14)	0.1998 (3)	0.0420 (6)	
C2	0.48749 (15)	0.65988 (16)	−0.0265 (3)	0.0349 (6)	
H2	0.4562	0.6989	−0.1008	0.042*	
C10	0.50824 (16)	0.57576 (17)	0.2313 (3)	0.0394 (6)	
H10	0.4887	0.5587	0.3331	0.047*	
C11	0.37390 (15)	0.66290 (14)	0.1614 (3)	0.0278 (5)	
C8	0.61064 (15)	0.57047 (16)	0.0508 (3)	0.0360 (6)	
C3	0.56531 (16)	0.62891 (17)	−0.0660 (3)	0.0374 (6)	
C7	0.68710 (17)	0.53586 (18)	0.0112 (4)	0.0471 (7)	
H7	0.7182	0.4980	0.0885	0.057*	
C4	0.59749 (19)	0.6509 (2)	−0.2199 (4)	0.0626 (9)	
H4	0.5687	0.6903	−0.2970	0.075*	
C6	0.71558 (19)	0.5574 (2)	−0.1385 (4)	0.0634 (9)	
H6	0.7659	0.5337	−0.1638	0.076*	
C5	0.6704 (2)	0.6145 (3)	−0.2557 (5)	0.0743 (11)	
H5	0.6904	0.6279	−0.3590	0.089*	
N1	0.00972 (12)	0.44091 (13)	0.2997 (3)	0.0352 (5)	
C22	0.15016 (13)	0.63803 (14)	0.3447 (3)	0.0244 (5)	
C12	0.11785 (14)	0.54892 (15)	0.3760 (3)	0.0272 (5)	
C21	0.04213 (15)	0.51849 (15)	0.2802 (3)	0.0323 (6)	
H21	0.0134	0.5556	0.1982	0.039*	
C13	0.16177 (15)	0.49416 (16)	0.4924 (3)	0.0319 (6)	
H13	0.2113	0.5129	0.5586	0.038*	
C18	0.02177 (17)	0.30129 (17)	0.4363 (4)	0.0423 (7)	
H18	−0.0301	0.2856	0.3752	0.051*	
C14	0.13216 (16)	0.40902 (15)	0.5126 (3)	0.0327 (6)	
C19	0.05460 (16)	0.38521 (15)	0.4152 (3)	0.0329 (6)	
C17	0.0653 (2)	0.24367 (19)	0.5447 (4)	0.0543 (8)	
H17	0.0433	0.1884	0.5569	0.065*	
C15	0.17626 (18)	0.34683 (18)	0.6242 (4)	0.0470 (7)	
H15	0.2280	0.3612	0.6876	0.056*	
C16	0.1433 (2)	0.26606 (19)	0.6392 (4)	0.0579 (9)	
H16	0.1727	0.2254	0.7126	0.069*	
O3	0.18843 (9)	0.67991 (10)	0.47024 (19)	0.0292 (4)	
O4	0.14006 (10)	0.66737 (10)	0.1932 (2)	0.0335 (4)	
O1	0.36040 (11)	0.66234 (11)	0.3145 (2)	0.0405 (4)	
O2	0.32086 (9)	0.69201 (10)	0.0404 (2)	0.0312 (4)	
O1W	0.15496 (10)	0.88329 (10)	0.3557 (2)	0.0360 (4)	
H1W	0.1487	0.8714	0.4588	0.054*	
H2W	0.1156	0.9051	0.3282	0.054*	
O2W	0.34752 (10)	0.88362 (11)	0.1592 (2)	0.0414 (5)	
H3W	0.3577	0.8785	0.0459	0.062*	
H4W	0.3751	0.9313	0.1964	0.062*	

### Atomic displacement parameters (Å^2^)

**Table:**

	*U*^11^	*U*^22^	*U*^33^	*U*^12^	*U*^13^	*U*^23^
Ca1	0.0245 (3)	0.0257 (2)	0.0204 (3)	−0.0003 (2)	0.00171 (18)	0.00021 (19)
C1	0.0283 (13)	0.0289 (13)	0.0266 (13)	0.0030 (10)	−0.0005 (10)	−0.0037 (10)
N2	0.0387 (13)	0.0447 (13)	0.0421 (14)	0.0147 (11)	0.0040 (10)	0.0051 (11)
C2	0.0309 (14)	0.0390 (14)	0.0332 (15)	0.0070 (11)	−0.0014 (11)	0.0048 (12)
C10	0.0383 (16)	0.0442 (16)	0.0357 (15)	0.0123 (12)	0.0049 (12)	0.0048 (12)
C11	0.0328 (14)	0.0238 (12)	0.0261 (14)	0.0012 (10)	0.0009 (11)	−0.0020 (10)
C8	0.0306 (15)	0.0365 (14)	0.0398 (16)	0.0041 (11)	0.0015 (12)	−0.0056 (12)
C3	0.0332 (15)	0.0424 (15)	0.0370 (15)	0.0036 (12)	0.0057 (12)	0.0014 (12)
C7	0.0353 (16)	0.0483 (17)	0.058 (2)	0.0118 (13)	0.0067 (14)	−0.0031 (14)
C4	0.051 (2)	0.086 (2)	0.056 (2)	0.0174 (17)	0.0216 (16)	0.0237 (18)
C6	0.0412 (19)	0.077 (2)	0.076 (2)	0.0149 (17)	0.0228 (17)	−0.006 (2)
C5	0.057 (2)	0.111 (3)	0.062 (2)	0.017 (2)	0.0312 (18)	0.017 (2)
N1	0.0315 (12)	0.0328 (12)	0.0405 (13)	−0.0059 (9)	0.0020 (10)	−0.0042 (10)
C22	0.0215 (12)	0.0274 (12)	0.0248 (13)	−0.0006 (10)	0.0051 (10)	−0.0045 (10)
C12	0.0283 (13)	0.0287 (12)	0.0250 (13)	−0.0030 (10)	0.0052 (10)	−0.0035 (10)
C21	0.0309 (14)	0.0326 (14)	0.0326 (14)	−0.0038 (11)	0.0016 (11)	−0.0008 (11)
C13	0.0314 (14)	0.0352 (14)	0.0291 (14)	−0.0056 (11)	0.0040 (11)	−0.0029 (11)
C18	0.0436 (17)	0.0392 (15)	0.0453 (18)	−0.0111 (13)	0.0105 (13)	−0.0021 (13)
C14	0.0363 (15)	0.0330 (14)	0.0293 (14)	−0.0026 (11)	0.0061 (11)	0.0002 (11)
C19	0.0351 (15)	0.0317 (14)	0.0336 (15)	−0.0060 (11)	0.0107 (11)	−0.0065 (11)
C17	0.072 (2)	0.0379 (16)	0.055 (2)	−0.0167 (15)	0.0147 (17)	0.0054 (14)
C15	0.0496 (18)	0.0432 (16)	0.0461 (18)	−0.0063 (14)	−0.0005 (14)	0.0062 (13)
C16	0.073 (2)	0.0407 (17)	0.058 (2)	−0.0011 (16)	0.0035 (17)	0.0175 (15)
O3	0.0333 (9)	0.0287 (9)	0.0246 (9)	−0.0058 (7)	0.0010 (7)	−0.0030 (7)
O4	0.0404 (10)	0.0370 (10)	0.0225 (9)	−0.0079 (8)	0.0019 (7)	0.0020 (7)
O1	0.0460 (11)	0.0530 (11)	0.0231 (10)	0.0156 (9)	0.0070 (8)	0.0015 (8)
O2	0.0275 (9)	0.0386 (10)	0.0263 (10)	0.0074 (7)	0.0000 (7)	0.0015 (7)
O1W	0.0361 (10)	0.0431 (10)	0.0285 (10)	0.0110 (8)	0.0035 (8)	0.0025 (8)
O2W	0.0474 (12)	0.0481 (11)	0.0292 (10)	−0.0211 (9)	0.0070 (8)	−0.0055 (8)

### Geometric parameters (Å, °)

**Table:**

Ca1—O3^i^	2.3829 (16)	C5—H5	0.9300
Ca1—O1W	2.3871 (16)	N1—C21	1.317 (3)
Ca1—O2^ii^	2.3877 (16)	N1—C19	1.371 (3)
Ca1—O2W	2.4202 (17)	C22—O4	1.254 (3)
Ca1—O4	2.4712 (16)	C22—O3	1.258 (3)
Ca1—O1	2.5404 (17)	C22—C12	1.495 (3)
Ca1—O2	2.5538 (16)	C12—C13	1.360 (3)
Ca1—O3	2.5689 (16)	C12—C21	1.413 (3)
Ca1—H1W	2.7828	C21—H21	0.9300
C1—C2	1.362 (3)	C13—C14	1.407 (3)
C1—C10	1.408 (3)	C13—H13	0.9300
C1—C11	1.496 (3)	C18—C17	1.350 (4)
N2—C10	1.318 (3)	C18—C19	1.410 (3)
N2—C8	1.368 (3)	C18—H18	0.9300
C2—C3	1.407 (3)	C14—C19	1.412 (3)
C2—H2	0.9300	C14—C15	1.414 (4)
C10—H10	0.9300	C17—C16	1.402 (4)
C11—O1	1.242 (3)	C17—H17	0.9300
C11—O2	1.261 (3)	C15—C16	1.360 (4)
C8—C3	1.407 (3)	C15—H15	0.9300
C8—C7	1.408 (3)	C16—H16	0.9300
C3—C4	1.410 (4)	O3—Ca1^ii^	2.3829 (16)
C7—C6	1.352 (4)	O2—Ca1^i^	2.3877 (16)
C7—H7	0.9300	O1W—H1W	0.8432
C4—C5	1.357 (4)	O1W—H2W	0.7195
C4—H4	0.9300	O2W—H3W	0.9231
C6—C5	1.395 (4)	O2W—H4W	0.8836
C6—H6	0.9300		
			
O3^i^—Ca1—O1W	86.63 (5)	O1—C11—C1	119.0 (2)
O3^i^—Ca1—O2^ii^	154.94 (6)	O2—C11—C1	118.7 (2)
O1W—Ca1—O2^ii^	79.95 (6)	N2—C8—C3	121.8 (2)
O3^i^—Ca1—O2W	75.13 (6)	N2—C8—C7	119.1 (2)
O1W—Ca1—O2W	97.97 (6)	C3—C8—C7	119.1 (3)
O2^ii^—Ca1—O2W	85.81 (5)	C2—C3—C8	117.8 (2)
O3^i^—Ca1—O4	78.80 (5)	C2—C3—C4	122.9 (2)
O1W—Ca1—O4	93.79 (6)	C8—C3—C4	119.1 (2)
O2^ii^—Ca1—O4	122.87 (5)	C6—C7—C8	120.3 (3)
O2W—Ca1—O4	150.61 (6)	C6—C7—H7	119.9
O3^i^—Ca1—O1	122.42 (5)	C8—C7—H7	119.9
O1W—Ca1—O1	150.78 (6)	C5—C4—C3	120.1 (3)
O2^ii^—Ca1—O1	74.02 (6)	C5—C4—H4	119.9
O2W—Ca1—O1	93.21 (6)	C3—C4—H4	119.9
O4—Ca1—O1	89.39 (6)	C7—C6—C5	120.9 (3)
O3^i^—Ca1—O2	71.54 (5)	C7—C6—H6	119.6
O1W—Ca1—O2	158.17 (6)	C5—C6—H6	119.6
O2^ii^—Ca1—O2	120.27 (6)	C4—C5—C6	120.5 (3)
O2W—Ca1—O2	76.99 (6)	C4—C5—H5	119.8
O4—Ca1—O2	82.10 (5)	C6—C5—H5	119.8
O1—Ca1—O2	50.97 (5)	C21—N1—C19	117.5 (2)
O3^i^—Ca1—O3	128.11 (6)	O4—C22—O3	122.3 (2)
O1W—Ca1—O3	82.58 (5)	O4—C22—C12	118.7 (2)
O2^ii^—Ca1—O3	71.20 (5)	O3—C22—C12	119.0 (2)
O2W—Ca1—O3	156.61 (6)	C13—C12—C21	118.4 (2)
O4—Ca1—O3	51.72 (5)	C13—C12—C22	121.1 (2)
O1—Ca1—O3	76.70 (5)	C21—C12—C22	120.5 (2)
O2—Ca1—O3	110.53 (5)	N1—C21—C12	124.2 (2)
O3^i^—Ca1—Ca1^i^	37.49 (4)	N1—C21—H21	117.9
O1W—Ca1—Ca1^i^	123.88 (4)	C12—C21—H21	117.9
O2^ii^—Ca1—Ca1^i^	151.60 (4)	C12—C13—C14	119.9 (2)
O2W—Ca1—Ca1^i^	76.36 (4)	C12—C13—H13	120.1
O4—Ca1—Ca1^i^	74.71 (4)	C14—C13—H13	120.1
O1—Ca1—Ca1^i^	84.96 (4)	C17—C18—C19	120.3 (3)
O2—Ca1—Ca1^i^	34.37 (4)	C17—C18—H18	119.9
O3—Ca1—Ca1^i^	122.74 (4)	C19—C18—H18	119.9
O3^i^—Ca1—Ca1^ii^	155.93 (4)	C13—C14—C19	117.7 (2)
O1W—Ca1—Ca1^ii^	75.75 (4)	C13—C14—C15	123.3 (2)
O2^ii^—Ca1—Ca1^ii^	37.14 (4)	C19—C14—C15	118.9 (2)
O2W—Ca1—Ca1^ii^	122.94 (4)	N1—C19—C18	118.5 (2)
O4—Ca1—Ca1^ii^	86.04 (4)	N1—C19—C14	122.3 (2)
O1—Ca1—Ca1^ii^	75.51 (4)	C18—C19—C14	119.2 (2)
O2—Ca1—Ca1^ii^	124.99 (4)	C18—C17—C16	120.9 (3)
O3—Ca1—Ca1^ii^	34.37 (3)	C18—C17—H17	119.5
Ca1^i^—Ca1—Ca1^ii^	152.71 (2)	C16—C17—H17	119.5
O3^i^—Ca1—H1W	102.1	C16—C15—C14	120.2 (3)
O1W—Ca1—H1W	16.6	C16—C15—H15	119.9
O2^ii^—Ca1—H1W	68.0	C14—C15—H15	119.9
O2W—Ca1—H1W	107.6	C15—C16—C17	120.5 (3)
O4—Ca1—H1W	90.8	C15—C16—H16	119.8
O1—Ca1—H1W	134.6	C17—C16—H16	119.8
O2—Ca1—H1W	171.3	C22—O3—Ca1^ii^	161.76 (15)
O3—Ca1—H1W	68.3	C22—O3—Ca1	89.47 (13)
Ca1^i^—Ca1—H1W	138.4	Ca1^ii^—O3—Ca1	108.15 (6)
Ca1^ii^—Ca1—H1W	59.2	C22—O4—Ca1	94.08 (13)
C2—C1—C10	118.0 (2)	C11—O1—Ca1	91.84 (14)
C2—C1—C11	121.0 (2)	C11—O2—Ca1^i^	160.71 (15)
C10—C1—C11	121.0 (2)	C11—O2—Ca1	90.76 (13)
C10—N2—C8	118.1 (2)	Ca1^i^—O2—Ca1	108.49 (6)
C1—C2—C3	120.2 (2)	Ca1—O1W—H1W	109.4
C1—C2—H2	119.9	Ca1—O1W—H2W	141.1
C3—C2—H2	119.9	H1W—O1W—H2W	99.8
N2—C10—C1	124.0 (2)	Ca1—O2W—H3W	116.8
N2—C10—H10	118.0	Ca1—O2W—H4W	139.0
C1—C10—H10	118.0	H3W—O2W—H4W	103.8
O1—C11—O2	122.2 (2)		

Symmetry codes: (i) *x*, −*y*+3/2, *z*−1/2; (ii) *x*, −*y*+3/2, *z*+1/2.

### Hydrogen-bond geometry (Å, °)

**Table:**

*D*—H···*A*	*D*—H	H···*A*	*D*···*A*	*D*—H···*A*
O2W—H4W···N2^iii^	0.88	2.01	2.880 (3)	170
O2W—H3W···O1^i^	0.92	1.92	2.813 (2)	163
O1W—H2W···N1^iv^	0.72	2.18	2.885 (2)	165
O1W—H1W···O4^ii^	0.84	1.94	2.785 (2)	174

Symmetry codes: (iii) −*x*+1, *y*+1/2, −*z*+1/2; (i) *x*, −*y*+3/2, *z*−1/2; (iv) −*x*, *y*+1/2, −*z*+1/2; (ii) *x*, −*y*+3/2, *z*+1/2.

## References

[bb1] Bruker (2008). *APEX2*, *SAINT* and *SADABS* Bruker AXS Inc., Madison, Wisconsin, USA.

[bb2] Haendler, H. M. (1986). *Acta Cryst.* C**42**, 147–149.

[bb3] Haendler, H. M. (1996). *Acta Cryst.* C**52**, 801–803.

[bb4] Hu, S., Zhang, S.-H. & Zeng, M.-H. (2007). *Acta Cryst.* E**63**, m2565.

[bb5] Martell, A. E. & Smith, R. M. (1974). *Critical Stability Constants*, Vol. 1, pp. 78, 372; Vol. 2, p. 219. New York: Plenum Press.

[bb6] Odoko, M., Muranishi, Y. & Okabe, N. (2001). *Acta Cryst.* E**57**, m267–m269.

[bb7] Okabe, N. & Koizumi, M. (1997). *Acta Cryst.* C**53**, 852–854.

[bb8] Okabe, N. & Makino, T. (1998). *Acta Cryst.* C**54**, 1279–1280.

[bb9] Okabe, N. & Makino, T. (1999). *Acta Cryst.* C**55**, 300–302.

[bb10] Okabe, N. & Muranishi, Y. (2002). *Acta Cryst.* E**58**, m287–m289.

[bb11] Sheldrick, G. M. (2008). *Acta Cryst.* A**64**, 112–122. 10.1107/S0108767307043930 18156677

